# Autophagy–lysosome pathway alterations and alpha-synuclein up-regulation in the subtype of neuronal ceroid lipofuscinosis, CLN5 disease

**DOI:** 10.1038/s41598-018-36379-z

**Published:** 2019-01-17

**Authors:** Jessie Adams, Melissa Feuerborn, Joshua A. Molina, Alexa R. Wilden, Babita Adhikari, Theodore Budden, Stella Y. Lee

**Affiliations:** 1 Division of Biology, Kansas State University, Manhattan, KS 66506 USA; 2 Present Address: Department of Cell Biology, Microbiology, and Molecular Biology, University of South Florida, Tampa, FL 33602 USA; 3 Present Address: University of Kansas School of Medicine, Kansas City, KS 66160 USA; 4 Present Address: Department of Orthopedic Surgery, University of Kansas Medical Center, Kansas City, KS 66160 USA

## Abstract

Neuronal ceroid lipofuscinoses (NCLs) are a group of inherited neurodegenerative lysosomal storage disorders. CLN5 deficiency causes a subtype of NCL, referred to as CLN5 disease. CLN5 is a soluble lysosomal protein with an unclear function in the cell. Increased levels of the autophagy marker protein LC3-II have been reported in several subtypes of NCLs. In this report, we examine whether autophagy is altered in CLN5 disease. We found that the basal level of LC3-II was elevated in both CLN5 disease patient fibroblasts and CLN5-deficient HeLa cells. Further analysis using tandem fluorescent mRFP-GFP-LC3 showed the autophagy flux was increased. We found the alpha-synuclein (α-syn) gene *SNCA* was highly up-regulated in CLN5 disease patient fibroblasts. The aggregated form of α-syn is well known for its role in the pathogenicity of Parkinson’s disease. Higher α-syn protein levels confirmed the *SNCA* up-regulation in both patient cells and CLN5 knockdown HeLa cells. Furthermore, α-syn was localized to the vicinity of lysosomes in CLN5 deficient cells, indicating it may have a lysosome-related function. Intriguingly, knocking down *SNCA* reversed lysosomal perinuclear clustering caused by CLN5 deficiency. These results suggest α-syn may affect lysosomal clustering in non-neuronal cells, similar to its role in presynaptic vesicles in neurons.

## Introduction

Neuronal ceroid lipofuscinoses (NCLs) are a group of progressive neurodegenerative lysosomal disorders that predominantly affect children^1,2^. There are thirteen genetically distinct subtypes of the NCLs that are named based on the genes in which the mutations have been identified^3^. Intriguingly, these genes encode a variety of unrelated proteins that are localized to various cellular compartments. Detrimental mutations in any of these genes cause the proteinaceous buildups of subunit C of the mitochondrial ATP synthase and/or saposin A and D in lysosomal compartments^4–6^. The similar phenotype associated with these mutations suggests that the NCL-related proteins are involved in a common cellular pathway or contribute to processes that are functionally linked, resulting in similar lysosomal dysfunction and waste accumulation.

Macroautophagy (hereafter referred to as autophagy) is part of the lysosomal degradation system. In contrast to the endocytic degradation pathway, the autophagy process brings intracellular material, such as long-lived cytosolic proteins and unwanted organelles, to lysosomes for disposal. The autophagy pathway is therefore inseparable from lysosome functions. Abnormal autophagy has been associated with several neurodegenerative diseases and lysosomal storage disorders^7–9^. In NCLs, an altered or impaired autophagy pathway has also been implicated. For example, higher basal levels of LC3-II, a marker for autophagosome formation, have been observed in murine models of various subtypes of NCL, including CLN2/TPP1^10^, CLN3^11^, CLN5^12^, CLN6^13^, CLN7^14^, and CLN10/cathepsin D^15^ diseases. On the other hand, reduced autophagy flux has been found in CLN5^−/−^ and CLN6^−/−^ ovine neural cultures^16^. This discrepancy of the latter study may be due to different cell types or animal models used in the studies.

In this report, we use CLN5-deficient NCL human patient skin fibroblasts and CLN5 knockdown (KD) HeLa cells to examine the autophagy-lysosome pathway. The CLN5 gene encodes a lysosomal luminal glycoprotein^17,18^. The function of CLN5 in lysosomes remains elusive. A possible role in endosomal sorting was suggested for human CLN5^19^. Another report suggests a CLN5 orthologue in *Dictyostelium* has glycoside hydrolase activity^20^. Here we show in CLN5-deficient cells the basal level of LC3-II is elevated, the autophagy flux is increased, and the expression level of α-syn gene *SNCA* is up-regulated. α-syn is highly expressed in presynaptic neurons and primarily localized to synaptic vesicles^21,22^. The presence of cytoplasmic inclusions filled with insoluble α-syn aggregates is a hallmark of Parkinson’s disease^23^. While α-syn has been implicated in several cellular processes, including synaptic vesicle endocytosis^24^ and exocytosis^25^, its exact function remains unclear. Despite being primarily associated with neurodegenerative disorders, both CLN5 and α-syn can be detected in a variety of tissues and cell types^26–31^. While this indicates more general roles of CLN5 and α-syn in non-neuronal tissues, there have been few studies performed to investigate these roles. The exogenously overexpressed α-syn has been shown to indirectly affect autophagy through the early secretory pathway protein Rab1a in cell culture systems^32^. Interestingly, we found the endogenous α-syn localizes to the lysosomes in human fibroblasts and HeLa cells. Furthermore, we uncovered a potential role for α-syn in regulating lysosomal positioning.

## Results

### Autophagy flux is increased in CLN5-deficient cells

**Figure 1:**
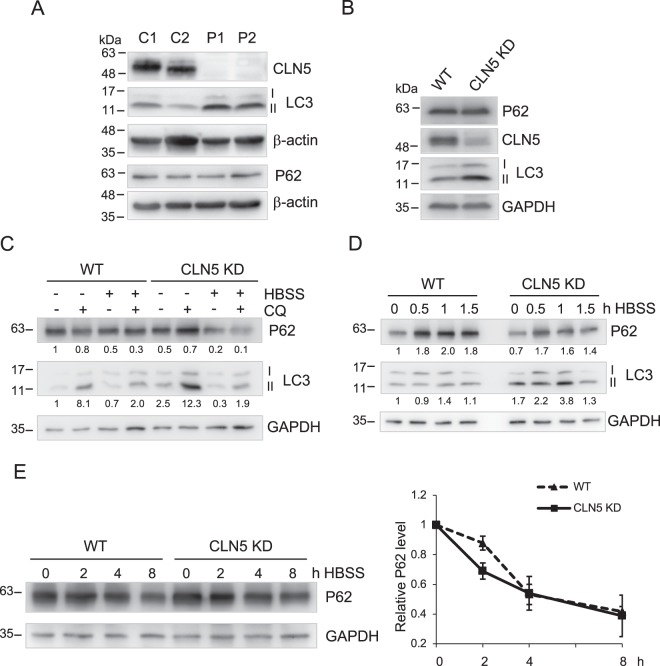
Autophagy is enhanced in CLN5-deficient cells. (**A**) Total lysates of two healthy control fibroblasts (C1 and C2) and two CLN5 disease patient fibroblasts (P1 and P2) were analyzed by immunoblotting. Basal levels of autophagic markers LC3-II and P62 were shown. The CLN5 was absent in patient cells. (**B**) Total lysates of WT and stable CLN5 KD HeLa cells were analyzed by immunoblotting. The CLN5 was greatly reduced in CLN5 KD cells. Basal levels of autophagic markers LC3-II and P62 were shown. (**C**) WT and Stable CLN5 KD HeLa cells were treated with CQ, HBSS, or HBSS + CQ for 4 h. Samples were analyzed by immunoblotting. The relative amounts of LC3-II and P62 after normalization with GAPDH are indicated. (**D**) WT and Stable CLN5 KD HeLa cells were incubated with HBSS for 0, 0.5, 1, 1.5 h. Samples were analyzed by immunoblotting. The relative amounts of LC3-II and P62 after normalization with GAPDH are indicated. (**E**) WT and Stable CLN5 KD HeLa cells were incubated with HBSS for 0, 2, 4, 8 h in the presence of cycloheximide and bortezomib. Samples were analyzed by immunoblotting. For degradation quantification on the right (N = 3), P62 was normalized with GAPDH signal in each lane. 0 h in each cell line was set as 1. Error bar represents SEM. β-actin and GAPDH were blotted as loading controls. All experiments were repeated at least three times.

As an initial step to examine whether the autophagy process might be altered with CLN5 deficiency, we compared the basal levels of an autophagy marker, LC3-II, in fibroblasts from control healthy individuals and fibroblasts derived from CLN5-deficient patients. LC3-II is a lipid-modified form of LC3 that is produced during autophagosome formation and is a commonly used readout for autophagy^33,34^. We found the protein level of LC3-II in CLN5-deficient patient fibroblasts was higher than in control cells (Fig. 1A). To ensure the effects observed were solely due to CLN5 deficiency in patient cells, we generated a CLN5 stable KD cell line with shRNA expression (Fig. 1B). The CLN5 protein level was dramatically reduced in CLN5 stable KD cells. Similar to CLN5-deficient patient cells, an increased LC3-II level was also observed in CLN5 KD HeLa cells. This is consistent with previous studies in various subtypes of NCLs^11–15^. When cells were treated with chloroquine (CQ) to block lysosomal degradation, there was a further accumulation of LC3-II in both wildtype and CLN5KD cells (Fig. 1C). This indicates that the higher basal levels of LC3-II seen in CLN5-deficient cells was not because the lysosome was unable to degrade LC3-II, but actually there was more LC3-II produced^34^.

Next, we examined the level of P62, an adaptor protein that links ubiquitinated cargo proteins to LC3-II. Upon autophagy activation, P62 is recruited to autophagosomes and eventually degraded in autolysosomes. On the other hand, accumulation of P62 upon autophagy activation (e.g. by starvation) indicates autophagy impairment. Therefore, the level of P62 is a commonly used measurement for autophagy flux^33^. Unlike LC3-II, we did not observe changes in the basal level of P62 in CLN5-deficient cells (Fig. 1A,B). Next, we followed P62 levels upon starvation to test if CLN5 deficiency blocks autophagic degradation (Fig. 1D,E). Within 1.5 h of starvation, P62 levels were increased in both WT and CLN5 KD HeLa cells (Fig. 1D), suggesting P62 was up-regulated^35^. We then followed long term starvation for up to 8 hours. Starvation was performed in the presence of cycloheximide (to block protein synthesis) and bortezomib (to block proteasome degradation). Under these conditions, P62 was degraded equally efficient in WT and CLN5 KD cells, indicating CLN5 deficient cells were capable of degrading autolysosome materials (Fig. 1E). Similar results were found in control and patient fibroblasts (Fig. S1).

**Figure 2:**
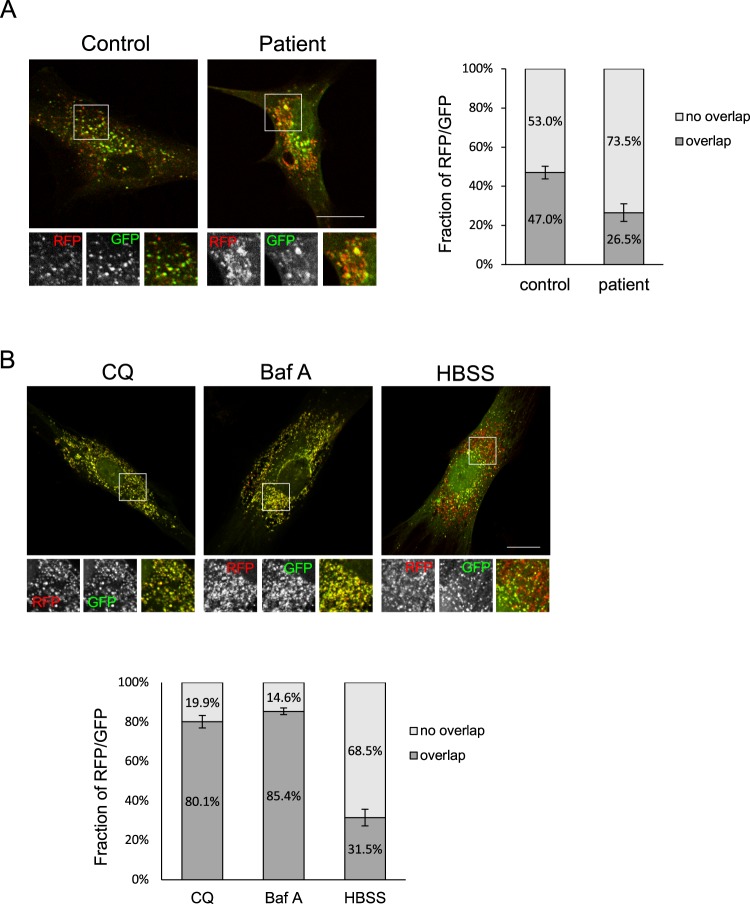
More autolysosomes are present in CLN5-deficient patient fibroblasts. (**A**) Control or CLN5-deficient fibroblasts that had been transfected with the mRFP-GFP-LC3 plasmid for 24 hours were fixed. GFP and RFP signal were visualized using confocal microscopy. The fraction of RFP/GFP signals that was overlapping was analyzed by the Mander’s coefficient using the ImageJ JACoP plugin. In ImageJ, two channels of each image file were first split to two separate images. These two images were then used for plugin JACoP analysis. The threshold of each image was adjusted to reduce the background signals before performing JACoP Mander’s coefficient. n = 15 cells, error bar represents SEM. (**B**) Control fibroblasts that had been transfected with the mRFP-GFP-LC3 plasmid for 24 hours were treated with CQ, baf A, or HBSS for 2 h prior to fixation. The fraction of RFP/GFP signals that was overlapping was analyzed by the Mander’s coefficient using the ImageJ JACoP plugin. n = 7 cells, error bar represents SEM. All experiments were repeated at least three times. Scale bar: 20 μm.

To further monitor the autophagy flux, we expressed mRFP-GFP-LC3 in human fibroblasts. The tandem fluorescent mRFP-GFP-LC3 has been used to follow the maturation progression of autolysosomes from autophagosomes, as the GFP fluorescence will be quenched in a more acidic environment such as autolysosomes, leaving the protein with only the mRFP signal^36^. Compared to control cells, the mRFP-GFP-LC3 emitted a stronger red fluorescent signal in CLN5-deficient cells (Fig. 2A). In addition, there were more punctate structures with only red fluorescence signals in CLN5 patient cells, suggesting more autolysosomes were present. The Mander’s coefficient was shown as a percentage of red fluorescent signal overlapping with green fluorescent signal (Fig. 2A, right). In patient cells the RFP signal was less overlapped with GFP signal. As control experiments, we also treated control fibroblasts with either lysosome inhibitors or starvation to examine the mRFP-GFP-LC3 response (Fig. 2B). In cells incubated with CQ or bafilomycin A (baf A), the overlaps between mRFP and GFP increased to ~80% (Fig. 2B), compared to no treatment of ~50% (Fig. 2A, control). On the other hand, when autophagy was induced by starvation (HBSS), the overlaps between mRFP and GFP was reduced to ~30%, similar to what we observed in patient cells. This supports our notion that cells lacking the CLN5 protein have higher basal levels of autophagic activity.

### Up-regulation of alpha-synuclein gene *SNCA* in CLN5-deficient cells

To further elucidate the changes of autophagy pathway in CLN5 disease patient cells, we performed quantitative PCR (qPCR) to follow a set of genes involved in the autophagy pathway (RT^2^ Profiler PCR array from SABiosciences). Table 1 lists the genes that were greater than two-fold up or down-regulated (with a p value < 0.05 from three repeats) in CLN5-deficient patient cells. Interestingly, none of the genes directly involved in autophagy initiation or machinery, e.g. *ATG* genes, were differentially expressed (data not shown). This suggests that the effects on autophagy flux with CLN5 deficiency are most likely through indirect regulation of the autophagy process. Among these genes, *SNCA* showed the greatest difference compared to control cells with ~90-fold increase in expression under normal cultured conditions. *SNCA* encodes α-syn, a protein well known for its role in the pathogenicity of Parkinson’s disease^23^.

**Table 1:** Autophagy pathway qPCR results of CLN5-deficient patient fibroblasts.

Gene Symbol	Fold Regulation	p-value
*SNCA*	+90.3923	0.0003
*CTSS*	+3.6365	0.0014
*BCL2*	−2.4529	0.0095
*HGS*	−2.3706	0.0065
*CASP3*	−2.3312	0.0008
*CDKN1B*	−2.1028	0.0002
*TGFB1*	−2.0955	0.0097
*PTEN*	−2.0499	0.0312

The genes with expression change (compared to control fibroblasts) >2-fold with a p-value < 0.05 are shown. Three independent qPCR were performed. +, up-regulated; −, down-regulated. The p values were calculated based on a Student’s t-test of the replicate 2^(−ΔCt)^ values for each gene in the control group and the CLN5 patient group.

**Figure 3:**
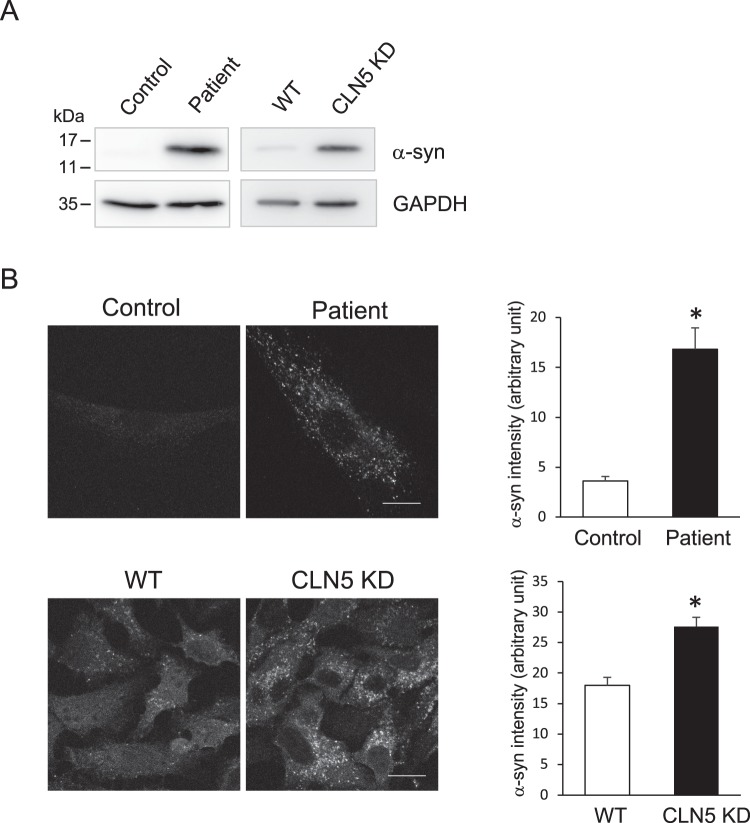
Higher levels of α-syn were detected in CLN5-deficient cells. (**A**) Total lysates from control and CLN5 disease patient fibroblasts, as well as WT and CLN5 KD HeLa cells were analyzed by immunoblotting. GAPDH was blotted as a loading control. (**B**) Control and CLN5 disease patient fibroblasts (top), as well as WT and CLN5 KD HeLa cells (bottom) were immunostained for α-syn. Signal intensity was quantified using ImageJ. Background was subtracted from the total intensity of each cell measured to obtain the correct total cell fluorescence. Data was analyzed using a Student’s t test with two-samples assuming equal variances. *P < 0.0005, n = 5 cells for fibroblasts; *P < 0.0005, n = 20 cells for WT and CLN5 KD HeLa cells, error bar represents SEM. Images were acquired using confocal microscopy. Scale bar: 20 μm. All experiments were repeated at least three times.

Immunoblotting was performed to verify the qPCR results. In normal fibroblasts, α-syn was below the detection level. On the other hand, we were able to detect α-syn expression in CLN5 disease patient fibroblasts (Fig. 3A). Compared to control fibroblasts, HeLa cells synthesized a basal level of α-syn that was readily detectable. In CLN5 stable knockdown cells, α-syn expression level was further increased. Using immunofluorescence microscopy, we were able to visualize α-syn in CLN5-deficient cells (Fig. 3B). Consistent with immunoblotting results, the intensity of α-syn was greatly enhanced in cells lacking CLN5. Stably knocking down CLN5 in HeLa cells recapitulated the increased LC3-II and α-syn levels, indicating that the effects we observed in CLN5 patient fibroblasts were indeed due to CLN5 deficiency. Knocking down CLN5 by transient transfection in control fibroblasts as well as SH-SY5Y cells, however, did not increase the level of α-syn (Fig. S2), suggesting that the effects we observed in CLN5 disease patient cells and CLN5 stable knockdown cells are a consequence of long-term deficiency of CLN5.

**Figure 4:**
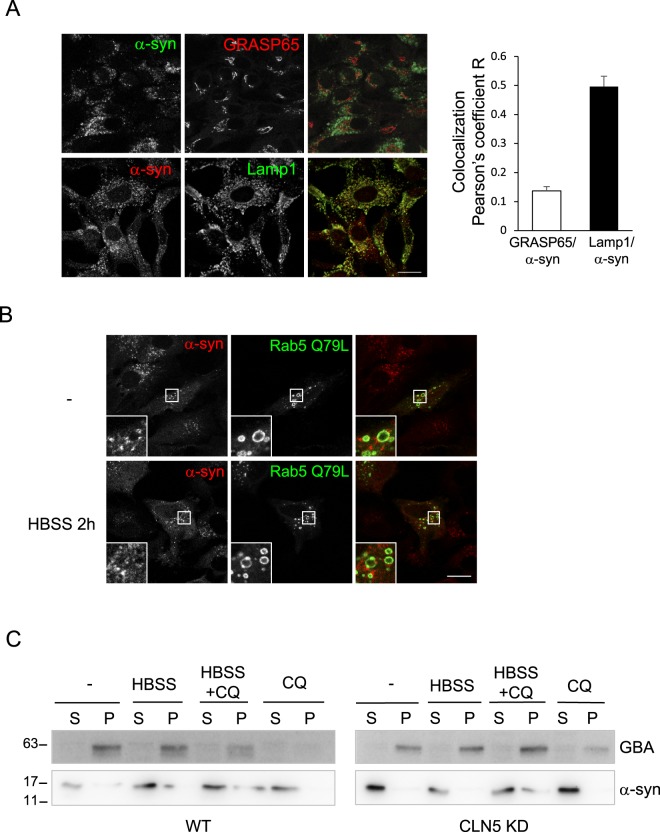
α-syn localizes to the vicinity of lysosomes. (**A**) CLN5 KD HeLa cells were fixed and immunostained for a Golgi marker, GRASP65 (top panel) and a lysosome marker, Lamp1 (bottom panel). The ImageJ JACoP plugin was used to analyze colocalization (Pearson’s coefficient). In ImageJ, two channels of each image file were first split to two separate images. These two images were then used for plugin JACoP analysis. Five images in each set (total 59 cells in GRASP/α-syn set; total 42 cells in Lamp1/α-syn set) were analyzed. Error bar represents SEM. (**B**) α-syn does not localize to the lumen of lysosomes. CLN5 KD HeLa cells that had been transfected with the EGFP-Rab5 Q79L plasmid for 24 hours were incubated with HBSS for 2 h prior to fixation and immunostained for α-syn. In control (−), cells were left untreated. Cells were fixed and immunostained for CLN5. Images were acquired using confocal microscopy. Scale bar: 20 μm. (**C**) Subcellular fractionation of α-syn. WT and Stable CLN5 KD HeLa cells were treated with CQ, HBSS, or HBSS + CQ for 2 h. After fractionation, samples were analyzed by immunoblotting. S, soluble; P, pellet. All experiments were repeated at least three times.

α-syn is an abundant protein in presynaptic neuronal cells and is associated with synaptic vesicles^21,22^. However, it can also be detected in a variety of tissues^27–31^. Upon exogenous overexpression, α-syn has been reported to localize to several different compartments^37–40^ and cause fragmentation of the Golgi apparatus^32,37^. Our finding of endogenously elevated expression levels of α-syn in CLN5-deficient cells prompted us to examine the localization of α-syn in these cells. We immunostained CLN5 KD cells with GRASP65 for the Golgi apparatus and Lamp1 for lysosomes, we found α-syn was largely colocalized with lysosomes but not the Golgi (Fig. 4A).

Several studies have suggested that α-syn can be degraded in the lysosomes via macroautophagy or the chaperone-mediated autophagy pathway^41–43^. Its accumulation has been observed in several lysosomal storage disorders (LSDs)^44–47^. Therefore, we subsequently analyzed if α-syn was localized in the lumen of the lysosomes as a substrate for degradation. In order to distinguish between outside and inside the lumen, we overexpressed GFP-Rab5 Q79L to induce enlarged endolysosomes^48^. Under normal conditions, endogenous α-syn was not present in the lumen (Fig. 4B, top), suggesting it is not subjected to degradation in lysosomes at the level of expression in CLN5-deficient cells. When cells were starved for 2 h to induce autophagy, no α-syn was found in the lumen of enlarged vacuoles either (Fig. 4B, bottom). Subcellular fractionation was performed to further analyze whether α-syn was indeed cytosolic under these conditions (Fig. 4C). β-glucosylceramidase (GBA) is a lysosomal luminal protein and was found in the “P” fraction, indicating the lysosomes in the membrane fraction were intact. In both WT and CLN5 KD HeLa cells, α-syn was found primarily in the soluble cytosolic fraction under normal conditions with or without CQ, indicating α-syn was not subjected to degradation. Interestingly, when cells were starved with HBSS for 2 h, a small fraction of α-syn was detected in the pellet fractions of both cell lines. This suggests α-syn can be degraded in the lysosomes when autophagy is induced. This is consistent with previous findings that α-syn can be degraded in the lysosomes via macroautophagy or the chaperone-mediated autophagy pathway^41–43^. Furthermore, there was no difference between WT and CLN5 KD cells in α-syn subcellular distribution, suggesting that increased α-syn in CLN5 deficient cells was not because of dysfunctional lysosomes.

### Alpha-synuclein and lysosomal clustering

**Figure 5:**
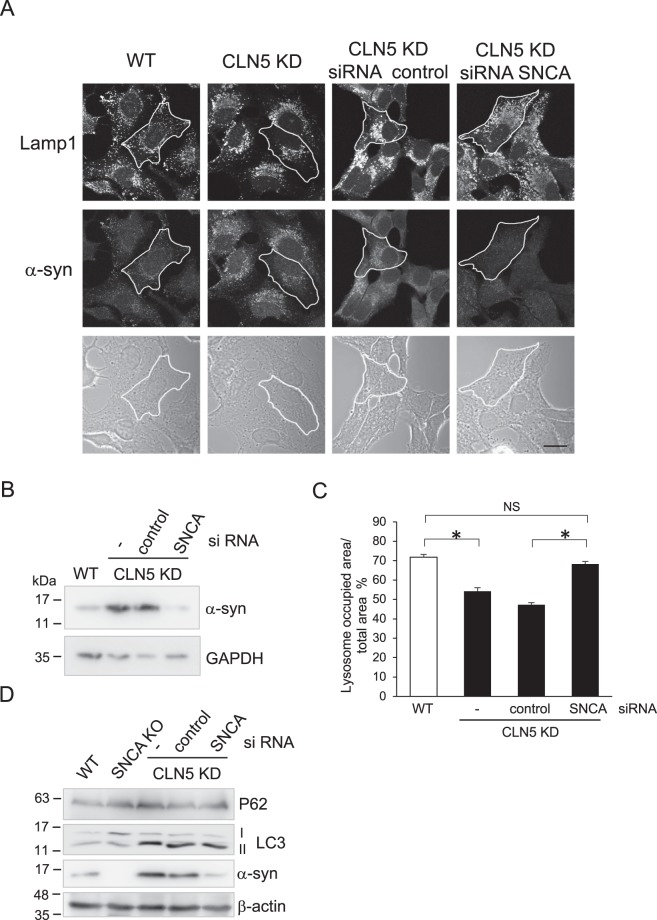
Knockdown of α-syn reverses the perinuclear lysosome clustering phenotype found in CLN5 KD HeLa cells. (**A**) HeLa or CLN5 KD cells were transfected with control or SNCA small interfering RNA (siRNA) for 48 hours prior to fixation. Lysosomes were visualized by immunostaining lamp1. Scale bar: 20 μm. (**B**) Immunoblot shows efficient knockdown of α-syn expression in CLN5 KD cells. GAPDH was blotted as a loading control. (**C**) The cellular distribution area of lysosomes was quantified in the conditions indicated. An outline of the cells was marked by tracing the cell border in phase contrast images. The total area of the cell and the area with lamp1 signal in the cell were measured using ImageJ “measure” function. One-way ANOVA followed by the Tukey’s post hoc test was performed *P < 0.05, n = 25, error bar represents SEM. (**D**) Immunoblotting of WT, stable SNCA knock-out cell (SNCA KO), and siRNA of stable CLN5 KD as indicated. β-actin was blotted as a loading control. All experiments were repeated at least three times.

The lysosomal localization of α-syn we observed reveals a possible function for α-syn in the lysosomal membrane. α-syn has a lipid binding property^49–51^ and has been shown to cause clustering of synaptic vesicles^52^. Therefore, we examined whether α-syn has any role in the lysosomal membrane in CLN5-deficient cells where *SNCA* is upregulated. Under normal growing conditions, the lysosomes are distributed throughout the cytoplasm. However, in some LSDs the lysosomes become perinuclear^53,54^. In CLN5 KD cells, the lysosomes were clustered to the perinuclear region as well (Fig. 5A). Remarkably, when we knocked down *SNCA* in CLN5 KD cells, the lysosomes were redistributed to the periphery of the cell (Fig. 5A). Figure 5A α-syn panel and 5B show efficient knockdown of *SNCA* in CLN5 KD cells. The quantification analysis showed that, in CLN5 KD cells, the lysosomal distribution area returned to the WT level when α-syn was reduced by siRNA (Fig. 5C). This suggests that α-syn plays a role in lysosomal clustering, a property similar to that found in synaptic vesicles^52^. We also examined the LC3 and P62 levels when we knocked down *SNCA* in CLN5 KD cells (Fig. 5D). Unlike its effect on lysosome position, transient *SNCA* knockdown did not change LC3-II in CLN5 KD cells back to the levels in WT or SNCA knockout cells. This suggests that the increase of autophagy flux in CLN5 deficient cells is not due to *SNCA* up-regulation.

## Discussion

Differential expression of α-syn has been shown in animal models of several NCL subtypes^47,55–58^. For example, accumulated α-syn has been found in CLN12 disease *Atp13a2*^−/−^ mouse model and *Atp13a2* knockdown mouse primary neurons^47,55^. On the other hand, a lower level of α-syn protein was found in the motor cortex of a naturally occurring CLN5-deficient sheep^56^. In addition, some mouse model studies have examined *Snca* gene expression. *Snca* expression was found to be substantially reduced in the cortex of *Cln1/Cln5* double knockout mice^57^, but not in *Cln1* or *Cln5* single knockout mice^57,59^. Yet in another study, *Snca* expression was highly up-regulated in the embryonic cortical neurons in *Cln1* knockout mice^58^. This highlights the complexity and variability of the regional and likely age-relevant expression.

In this study, we showed increased autophagy flux in CLN5-deficient cells by employing different measurements to monitor autophagy flux. In particular, the tandem fluorescent mRFP-GFP-LC3 assay demonstrated a higher basal autophagy flux in CLN5-deficient cells. The level of autophagy flux was similar to that of starvation induced autophagy in control cells. Since CLN5 disease is a lysosomal storage disorder, it is reasonable to assume increased autophagy is a compensatory effect in CLN5-deficient cells. Our data showed that lysosomal degradation of P62 was normal in CLN5-deficient cells, suggesting that CLN5 deficiency disrupts lysosome function other than lysosomal proteolysis.

Using PCR array of the autophagy pathway to analyze differential expression, we identified the *SNCA* gene as one of the autophagy-related genes up-regulated in CLN5 disease patient cells. Whether and how up-regulation of *SNCA* affects the autophagy process in CLN5-deficient cells remain to be determined. However, transient knocking down *SNCA* by siRNA in CLN5 KD cells did not reduce the LC3-II back to the level in WT cells, suggesting an indirect role of α-syn in regulating this process. Furthermore, while we observed up-regulation of the *SNCA* gene in CLN5 disease patient fibroblasts and stable CLN5 KD cells, transient CLN5 KD by siRNA in either fibroblasts or SH-SY5Y cells showed no up-regulation. These results suggest that *SNCA* up-regulation is a consequence of long-term CLN5 deficiency. A stable knockdown or knockout of CLN5 in SH-SY5Y cells or induced pluripotent stem cell (iPSC)-differentiated neuronal cells will help to elucidate whether *SNCA* up-regulation has a physiological relevance in NCL diseases.

The potential role of α-syn in lysosomal clustering we observed suggests a possible function of α-syn exists in non-neuronal cells. Using *in vitro* assays, it was shown that by binding to anionic lipid and synaptobrevin-2, a synaptic vesicle SNARE protein, α-syn caused clustering of the synaptobrevin-2 containing vesicles^25^. The clustering property of α-syn demonstrated herein is in agreement with the previous literature. For example, overexpression of α-syn inhibits synaptic vesicle release, possibly due to a reduced pool of readily releasable synaptic vesicles^60,61^. Similarly, deletion of α-syn increases neurotransmitter release^62^. It will be interesting to examine whether α-syn binds to any lysosomal SNARE proteins and whether the clustering effects of elevated levels of α-syn relate to the lysosome function in CLN5-deficient NCL and other NCLs in general.

Besides α-syn, defects in several proteins involved in endo-lysosomal trafficking, such as retromer subunit VPS35, leucine-rich repeat kinase 2 (LRRK2), Rab7L1, and Rab39B, are known to cause Parkinson’s disease^63–68^. Supporting a role of α-syn in the endo-lysosome pathway, it has been documented that in cortical neurons α-syn localizes to the vicinity of endocytic compartments and interacts directly with VPS29, SNX1 (two retromer subunits), and Rab4A (an endosomal Rab GTPase)^69^. Whether our finding of α-syn affecting lysosome positioning in HeLa cells is physiologically relevant in neurons and contributes to pathogenicity of Parkinson’s disease will need to be further examined.

## Materials and Methods

### Cell culture and transfections

Cell culture media and reagents were purchased from Gibco and Hyclone. Healthy control human fibroblasts were purchased from Coriell Cell Repositories (C1: GM05757 and C2: GM00498). CLN5 disease patient fibroblasts (P1: c.671G > A, p. Trp224X and exon 4 deletion; P2: homozygous c.694C > T, p. Glm232X) were received from Massachusetts General Hospital CHGR NCL Disorders Clinical Database and Biorepository. HeLa cells (ATCC CCL-2) and human fibroblasts were grown and maintained in Dulbecco’s modified eagle medium (DMEM) supplemented with 10% fetal bovine serum, 2 mM glutamine, 20 mM HEPES, and gentamicin at 37 °C in a humidified incubator with 5% CO_2_. 50 μM CQ (MP Biomedicals) and 20 nM baf A (Cayman Chemicals), 50 μg/ml cycloheximide (Fisher Scientific), and 100 nM bortezomib (LC Laboratories) were used in the treatments. The CLN5 stable knockdown cell line was created by transfecting a plasmid containing shRNA sequence against CLN5 (SABiosciences) to HeLa cells and selecting for resistance to hygromycin. The SNCA knock-out HeLa cell line was generated using CRISPR/Cas9 method (plasmid was purchased from GeneCopoeia). The control siRNA, siRNA for SNCA (SMARTpool), and siRNA for CLN5 (GAACCUACUUAUCUGGGAA) were purchased from Dharmacon. RNAiMAX (Thermo Fisher) was used for siRNA transfections in HeLa and SH-SY5Y cells. The Neon electroporation transfection system was used for siRNA transfection in fibroblasts. For overexpression transfections, the TransIT-LT1 transfection reagent was used (Mirus Bio). The tandem mRFP-GFP-LC3 plasmid ptfLC3 was a gift from Tamotsu Yoshimori (Addgene plasmid #21074)^36^. The EGFP-Rab5 Q79L plasmid was a gift from Qing Zhong (Addgene plasmid #28046)^70^.

### Quantitative polymerase chain reaction (qPCR)

RT^2^ Profiler PCR Array of the human autophagy pathway was purchased from SABiosciences. RNA was isolated from fresh fibroblast pellets using RNeasy Mini Kit (Qiagen). 0.5 μg of RNA was used for the first strand cDNA synthesis (RT^2^ First Strand Kit, SABiosciences). The cDNAs synthesized were used for RT^2^ Profiler PCR Array with RT^2^ SYBR Green qPCR Master Mixes samples. Three independent RNA prep samples were used for triplicate qPCR. Data was analyzed using RT^2^ Profiler PCR Array data analysis tool. Fold-Change (2^(−ΔΔCt)^) is the normalized gene expression (2^(−ΔCt)^) in the CLN5-deficient fibroblast sample divided by the normalized gene expression (2^(−ΔCt)^) of the control sample. The p values were calculated based on a Student’s t-test of the replicate 2^(−ΔCt)^ values for each gene in the control group and the CLN5 patient group.

### Sample preparation and Immunoblotting

Cells grown on culture dishes were scraped and washed once with 1X phosphate buffered saline (PBS), and centrifuged for 3 min at 1,500 × g. Cell pellets were lysed using RIPA lysis buffer (50 mM Tris pH 8.0, 150 mM NaCl, 1% NP-40, 0.5% sodium deoxycholate, 0.1% SDS) supplemented with protease inhibitor cocktail (G-Biosciences). After incubation for 30 min on ice, extracts were centrifuged at 20,000 × g for 10 min at 4 °C. The supernatant was collected as the whole cell lysates. Protein concentrations were determined by Bradford assay. Protein samples were separated by SDS-PAGE and transferred to PVDF membranes (Millipore) followed by immunoblotting. For the membranes that were prepared to blot for α-syn, after transfer, the membranes were first fixed with 1% formaldehyde in PBS for 30 min followed by blocking in 5% non-fat milk in TBST. ECL detection was performed according to the manufacturer’s instructions (Millipore) and blots were imaged using G-Box (Syngene). Quantification was performed using ImageJ.

### Immunofluorescence Microscopy

Cells on coverslips were fixed with 4% formaldehyde for 10 min at room temperature or 100% methanol for 15 min at −20 °C. Blocking, permeabilization, antibody incubations, and washes were done using blocking solution (10% fetal calf serum, 0.1% saponin, and 0.02% sodium azide in PBS). The cells were imaged using either Zeiss LSM-5 PASCAL or LSM-700 laser scanning confocal microscope. Quantification was performed using ImageJ. For comparison between groups, data was analyzed using a Student’s t test with two-samples assuming equal variances. For comparison among multiple groups, a one-way ANOVA followed by the Tukey’s post hoc test was performed.

### Subcellular fractionation

After treatments, cell pellets were collected, resuspended in isotonic buffer (10 mM HEPES, 10 mM KCl, 1 mM EDTA, 250 mM sucrose, 1 mM DTT, protease inhibitor cocktail), and incubated 10 min on ice. Samples were passed through a 25 G needle with 14 strokes. The post nuclear supernatant (PNS) was collected after centrifugation at 600 × g for 10 min at 4 °C. The PNS was centrifuged at 20,000 × g for 20 min at 4 °C. The supernatant was collected as the “soluble” fraction. The pellet was rinsed once with isotonic buffer and centrifuged again at 20,000 × g for 5 min at 4 °C. The pellet was resuspended in RIPA buffer as the “pellet” fraction. This is the crude membrane fraction that contains organelles (except nucleus and microsomes).

### Antibodies

Rabbit polyclonal antibodies used in this study were against LC3B (Abcam, ab51520), and GRASP65 (Pierce, PA3-910). Rabbit monoclonal antibodies used in this study were against CLN5 (Abcam, ab170899) and α-synuclein (Abcam, MJFR1). Mouse monoclonal antibodies used in this study were against β-actin (GenScript, A00702), GAPDH (Developmental Studies Hybridoma Bank, DSHB-hGAPDH-4B7, deposited by DSHB), α-synuclein (Abcam, 4D6), P62 (Abcam, ab56416), GBA (Novus, MAB7410), and Lamp1 (DSHB, G1/139/5, deposited by Hauri, H.-P.)^71^. HRP-conjugated secondary antibodies for Western blotting were purchased from Jackson Laboratory. Secondary antibodies conjugated to Alexa Fluor 488 or 546 were purchased from Molecular Probes.

## Electronic supplementary material

Supplementary information

## Data Availability

The datasets generated during and/or analyzed during the current study are available from the corresponding author on reasonable request.

## References

[1] Bennett MJ, Hofmann SL (1999). The neuronal ceroid-lipofuscinoses (Batten disease): a new class of lysosomal storage diseases. J Inherit Metab Dis.

[2] Haltia M (2006). The neuronal ceroid-lipofuscinoses: from past to present. Biochim Biophys Acta.

[3] Williams RE, Mole SE (2012). New nomenclature and classification scheme for the neuronal ceroid lipofuscinoses. Neurology.

[4] Palmer DN (1992). Mitochondrial ATP synthase subunit c storage in the ceroid-lipofuscinoses (Batten disease). Am J Med Genet.

[5] Tyynela J, Palmer DN, Baumann M, Haltia M (1993). Storage of saposins A and D in infantile neuronal ceroid-lipofuscinosis. FEBS Lett.

[6] Herva R, Tyynela J, Hirvasniemi A, Syrjakallio-Ylitalo M, Haltia M (2000). Northern epilepsy: a novel form of neuronal ceroid-lipofuscinosis. Brain Pathol.

[7] Settembre C (2008). A block of autophagy in lysosomal storage disorders. Hum Mol Genet.

[8] Raben N, Shea L, Hill V, Plotz P (2009). Monitoring autophagy in lysosomal storage disorders. Methods Enzymol.

[9] Wong E, Cuervo AM (2010). Autophagy gone awry in neurodegenerative diseases. Nat Neurosci.

[10] Micsenyi MC, Sikora J, Stephney G, Dobrenis K, Walkley SU (2013). Lysosomal membrane permeability stimulates protein aggregate formation in neurons of a lysosomal disease. J Neurosci.

[11] Cao Y (2006). Autophagy is disrupted in a knock-in mouse model of juvenile neuronal ceroid lipofuscinosis. J Biol Chem.

[12] Leinonen H (2017). Retinal Degeneration In A Mouse Model Of CLN5 Disease Is Associated With Compromised Autophagy. Sci Rep.

[13] Thelen M (2012). Disruption of the autophagy-lysosome pathway is involved in neuropathology of the nclf mouse model of neuronal ceroid lipofuscinosis. Plos One.

[14] Brandenstein L, Schweizer M, Sedlacik J, Fiehler J, Storch S (2016). Lysosomal dysfunction and impaired autophagy in a novel mouse model deficient for the lysosomal membrane protein Cln7. Hum Mol Genet.

[15] Koike M (2005). Participation of autophagy in storage of lysosomes in neurons from mouse models of neuronal ceroid-lipofuscinoses (Batten disease). Am J Pathol.

[16] Best HL (2017). Characterisation of early changes in ovine CLN5 and CLN6 Batten disease neural cultures for the rapid screening of therapeutics. Neurobiol Dis.

[17] Vesa J (2002). Neuronal ceroid lipofuscinoses are connected at molecular level: interaction of CLN5 protein with CLN2 and CLN3. Mol Biol Cell.

[18] Moharir A, Peck SH, Budden T, Lee SY (2013). The role of N-glycosylation in folding, trafficking, and functionality of lysosomal protein CLN5. Plos One.

[19] Mamo A, Jules F, Dumaresq-Doiron K, Costantino S, Lefrancois S (2012). The role of ceroid lipofuscinosis neuronal protein 5 (CLN5) in endosomal sorting. Mol Cell Biol.

[20] Huber RJ, Mathavarajah S (2017). Cln5 is secreted and functions as a glycoside hydrolase in Dictyostelium. Cell Signal.

[21] Maroteaux L, Campanelli JT, Scheller RH (1988). Synuclein: a neuron-specific protein localized to the nucleus and presynaptic nerve terminal. J Neurosci.

[22] Iwai A (1995). The precursor protein of non-A beta component of Alzheimer’s disease amyloid is a presynaptic protein of the central nervous system. Neuron.

[23] Spillantini MG (1997). Alpha-synuclein in Lewy bodies. Nature.

[24] Vargas KJ (2014). Synucleins regulate the kinetics of synaptic vesicle endocytosis. J Neurosci.

[25] Burre J (2010). Alpha-synuclein promotes SNARE-complex assembly *in vivo* and *in vitro*. Science.

[26] De Silva B, Adams J, Lee SY (2015). Proteolytic processing of the neuronal ceroid lipofuscinosis related lysosomal protein CLN5. Exp Cell Res.

[27] Ltic S (2004). Alpha-synuclein is expressed in different tissues during human fetal development. J Mol Neurosci.

[28] Askanas V, Engel WK, Alvarez RB, McFerrin J, Broccolini A (2000). Novel immunolocalization of alpha-synuclein in human muscle of inclusion-body myositis, regenerating and necrotic muscle fibers, and at neuromuscular junctions. J Neuropathol Exp Neurol.

[29] Shin EC (2000). Expression patterns of alpha-synuclein in human hematopoietic cells and in Drosophila at different developmental stages. Mol Cells.

[30] Nakai M (2007). Expression of alpha-synuclein, a presynaptic protein implicated in Parkinson’s disease, in erythropoietic lineage. Biochem Biophys Res Commun.

[31] Ueda K, Saitoh T, Mori H (1994). Tissue-dependent alternative splicing of mRNA for NACP, the precursor of non-A beta component of Alzheimer’s disease amyloid. Biochem Biophys Res Commun.

[32] Winslow AR (2010). alpha-Synuclein impairs macroautophagy: implications for Parkinson’s disease. J Cell Biol.

[33] Klionsky DJ (2016). Guidelines for the use and interpretation of assays for monitoring autophagy (3rd edition). Autophagy.

[34] Mizushima N, Yoshimori T (2007). How to interpret LC3 immunoblotting. Autophagy.

[35] Sahani MH, Itakura E, Mizushima N (2014). Expression of the autophagy substrate SQSTM1/p62 is restored during prolonged starvation depending on transcriptional upregulation and autophagy-derived amino acids. Autophagy.

[36] Kimura S, Noda T, Yoshimori T (2007). Dissection of the autophagosome maturation process by a novel reporter protein, tandem fluorescent-tagged LC3. Autophagy.

[37] Gosavi N, Lee HJ, Lee JS, Patel S, Lee SJ (2002). Golgi fragmentation occurs in the cells with prefibrillar alpha-synuclein aggregates and precedes the formation of fibrillar inclusion. J Biol Chem.

[38] Cooper AA (2006). Alpha-synuclein blocks ER-Golgi traffic and Rab1 rescues neuron loss in Parkinson’s models. Science.

[39] Thayanidhi N (2010). Alpha-synuclein delays endoplasmic reticulum (ER)-to-Golgi transport in mammalian cells by antagonizing ER/Golgi SNAREs. Mol Biol Cell.

[40] Nakamura K (2011). Direct membrane association drives mitochondrial fission by the Parkinson disease-associated protein alpha-synuclein. J Biol Chem.

[41] Webb JL, Ravikumar B, Atkins J, Skepper JN, Rubinsztein DC (2003). Alpha-Synuclein is degraded by both autophagy and the proteasome. J Biol Chem.

[42] Vogiatzi T, Xilouri M, Vekrellis K, Stefanis L (2008). Wild type alpha-synuclein is degraded by chaperone-mediated autophagy and macroautophagy in neuronal cells. J Biol Chem.

[43] Batelli S, Peverelli E, Rodilossi S, Forloni G, Albani D (2011). Macroautophagy and the proteasome are differently involved in the degradation of alpha-synuclein wild type and mutated A30P in an *in vitro* inducible model (PC12/TetOn). Neuroscience.

[44] Mazzulli JR (2011). Gaucher disease glucocerebrosidase and alpha-synuclein form a bidirectional pathogenic loop in synucleinopathies. Cell.

[45] Choi JH (2011). Aggregation of alpha-synuclein in brain samples from subjects with glucocerebrosidase mutations. Mol Genet Metab.

[46] Kang S, Heo TH, Kim SJ (2014). Altered levels of alpha-synuclein and sphingolipids in Batten disease lymphoblast cells. Gene.

[47] Usenovic M, Tresse E, Mazzulli JR, Taylor JP, Krainc D (2012). Deficiency of ATP13A2 leads to lysosomal dysfunction, alpha-synuclein accumulation, and neurotoxicity. J Neurosci.

[48] Stenmark H (1994). Inhibition of rab5 GTPase activity stimulates membrane fusion in endocytosis. EMBO J.

[49] Pranke IM (2011). alpha-Synuclein and ALPS motifs are membrane curvature sensors whose contrasting chemistry mediates selective vesicle binding. J Cell Biol.

[50] Middleton ER, Rhoades E (2010). Effects of curvature and composition on alpha-synuclein binding to lipid vesicles. Biophys J.

[51] Westphal CH, Chandra SS (2013). Monomeric synucleins generate membrane curvature. J Biol Chem.

[52] Diao J (2013). Native alpha-synuclein induces clustering of synaptic-vesicle mimics via binding to phospholipids and synaptobrevin-2/VAMP2. Elife.

[53] Li X (2016). A molecular mechanism to regulate lysosome motility for lysosome positioning and tubulation. Nat Cell Biol.

[54] Uusi-Rauva K (2012). Neuronal ceroid lipofuscinosis protein CLN3 interacts with motor proteins and modifies location of late endosomal compartments. Cell Mol Life Sci.

[55] Schultheis PJ (2013). Atp13a2-deficient mice exhibit neuronal ceroid lipofuscinosis, limited alpha-synuclein accumulation and age-dependent sensorimotor deficits. Hum Mol Genet.

[56] Amorim IS (2015). Molecular neuropathology of the synapse in sheep with CLN5 Batten disease. Brain Behav.

[57] Blom T (2013). Exacerbated neuronal ceroid lipofuscinosis phenotype in Cln1/5 double-knockout mice. Dis Model Mech.

[58] Ahtiainen L (2007). Palmitoyl protein thioesterase 1 (Ppt1)-deficient mouse neurons show alterations in cholesterol metabolism and calcium homeostasis prior to synaptic dysfunction. Neurobiol Dis.

[59] Kopra O (2004). A mouse model for Finnish variant late infantile neuronal ceroid lipofuscinosis, CLN5, reveals neuropathology associated with early aging. Human molecular genetics.

[60] Nemani VM (2010). Increased expression of alpha-synuclein reduces neurotransmitter release by inhibiting synaptic vesicle reclustering after endocytosis. Neuron.

[61] Larsen KE (2006). Alpha-synuclein overexpression in PC12 and chromaffin cells impairs catecholamine release by interfering with a late step in exocytosis. J Neurosci.

[62] Anwar S (2011). Functional alterations to the nigrostriatal system in mice lacking all three members of the synuclein family. J Neurosci.

[63] Zimprich A (2011). A mutation in VPS35, encoding a subunit of the retromer complex, causes late-onset Parkinson disease. Am J Hum Genet.

[64] Vilarino-Guell C (2011). VPS35 mutations in Parkinson disease. Am J Hum Genet.

[65] Zimprich A (2004). Mutations in LRRK2 cause autosomal-dominant parkinsonism with pleomorphic pathology. Neuron.

[66] Paisan-Ruiz C (2004). Cloning of the gene containing mutations that cause PARK8-linked Parkinson’s disease. Neuron.

[67] Wilson GR (2014). Mutations in RAB39B cause X-linked intellectual disability and early-onset Parkinson disease with alpha-synuclein pathology. Am J Hum Genet.

[68] MacLeod DA (2013). RAB7L1 interacts with LRRK2 to modify intraneuronal protein sorting and Parkinson’s disease risk. Neuron.

[69] Chung CY (2017). *In Situ* Peroxidase Labeling and Mass-Spectrometry Connects Alpha-Synuclein Directly to Endocytic Trafficking and mRNA Metabolism in Neurons. Cell Syst.

[70] Sun Q, Westphal W, Wong KN, Tan I, Zhong Q (2010). Rubicon controls endosome maturation as a Rab7 effector. Proc Natl Acad Sci USA.

[71] Schweizer A, Fransen JA, Bachi T, Ginsel L, Hauri HP (1988). Identification, by a monoclonal antibody, of a 53-kD protein associated with a tubulo-vesicular compartment at the cis-side of the Golgi apparatus. J Cell Biol.

