# Supplementary material for: Autophagy–lysosome pathway alterations and alpha-synuclein up-regulation in the subtype of neuronal ceroid lipofuscinosis, CLN5 disease
Source: Sci Rep. 2019 Jan 17;9:151. doi: 10.1038/s41598-018-36379-z (PMC6336884; doi:10.1038/s41598-018-36379-z)

Autophagy–lysosome pathway alterations and alpha-synuclein up-regulation in the subtype of neuronal ceroid lipofuscinosis, CLN5 disease

Jessie Adams[1§], Melissa Feuerborn[1§], Joshua A. Molina[1§], Alexa R. Wilden[1], Babita Adhikari[1], Theodore Budden[1], and Stella Y. Lee[1*]

[1] Division of Biology, Kansas State University, Manhattan, KS 66506, USA

[§] These authors contributed equally

[*] Corresponding author: Stella Y. Lee, sylee@ksu.edu

Supplementary information

Figure S1. CLN5 disease patient cells are capable of degrading P62 via lysosomes. WT and Stable CLN5 KD HeLa cells were incubated with HBSS for 0, 2, 4, 8 h in the presence of cycloheximide and bortezomib. Samples were analyzed by immunoblotting. β-actin was blotted as a loading control. For degradation quantification (N=3), P62 was normalized with β-actin signal in each lane. 0 h in each cell line was set as 1. Error bar represents SEM.

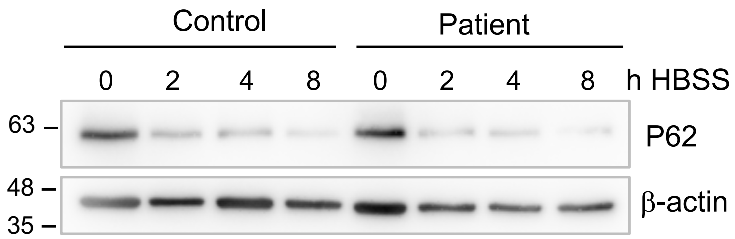

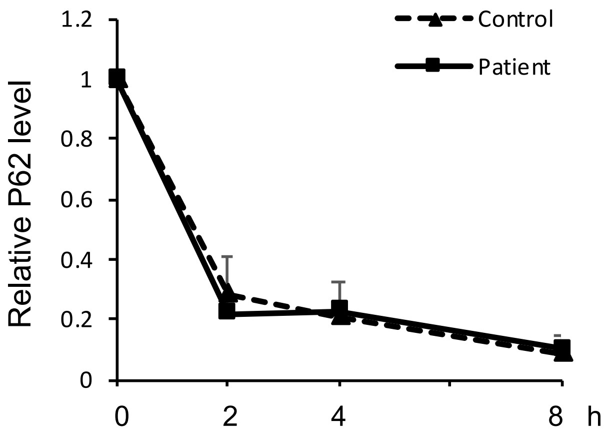

Figure S2. α-syn is not up-regulated by transient knockdown of CLN5. (A) Human fibroblasts were transfected with siRNA against CLN5 or control for 72 or 90 h as indicated. C: control fibroblasts; P: Patient fibroblasts. (B) SH-SY5Y cells were transfected with siRNA against CLN5 or control for 72 h. Samples were analyzed by immunoblotting. GAPDH was blotted as a loading control.

A

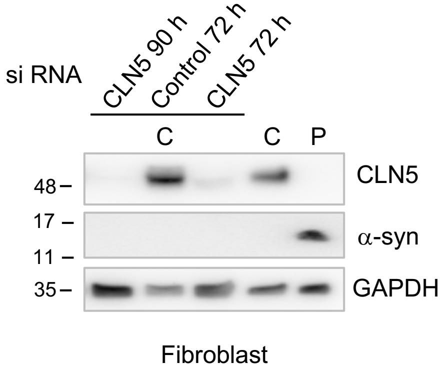

B

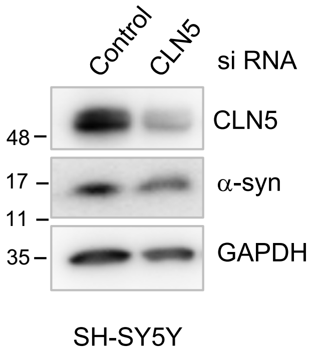

Supplement: Supplementary file 1 — Supplementary information [file 41598_2018_36379_MOESM1_ESM.pdf]
